# Integrating Free-Form Nanostructured GRIN Microlenses with Single-Mode Fibers for Optofluidic Systems

**DOI:** 10.1038/s41598-018-23464-6

**Published:** 2018-03-22

**Authors:** Rafal Kasztelanic, Adam Filipkowski, Alicja Anuszkiewicz, Paulina Stafiej, Grzegorz Stepniewski, Dariusz Pysz, Konrad Krzyzak, Ryszard Stepien, Mariusz Klimczak, Ryszard Buczynski

**Affiliations:** 1 Department of Glass, Institute of Electronic Materials Technology, Wolczynska 133, 01-919 Warsaw, Poland; 2 Faculty of Physics, University of Warsaw, Pasteura 7, 02-093 Warsaw, Poland

## Abstract

We present both a theoretical and an experimental study of a novel compact lensed fiber system utilizing a nanostructured GRIN lens. The lens can be integrated with an optical fiber, which ensures a unique and efficient focusing in any high index medium, such as a liquid. We use the effective medium approach to design lenses with arbitrary refractive index. To fabricate lenses, we utilize a discrete array of nano-sized rods made of two types of glasses, and apply a standard stack-and-draw fiber drawing technology. The fabricated nanostructured GRIN lenses have a parabolic refractive index profile with a diameter of a standard fiber, very short working distances (55 µm in the air) and a high numerical aperture (NA = 0.16). As a proof-of-concept of the new micro-lensed fiber system, we demonstrate an experiment on optical trapping of micrometer-sized glass beads. We also show that our method is compatible with optical fiber technology and allows for any shape of the refractive index distribution in 2D. Thanks to that a new functionality could be achieved by replacing the GRIN lens with an axicon lens, vortex type elements, micro-lenses arrays or diffraction elements.

## Introduction

The technology of optical manipulation and trapping of microscopic particles^1^ has been developed since the beginning of 1970s and has resulted in designing of numerous types of optical traps^2^. Most of them operate within a setup of an inverted microscope, where the optical trap is created by an objective of a large numerical aperture (NA), which strongly focuses the light beam. Additionally, setups of this kind often utilize spatial light modulators^3^, or deflected mirrors^4^ in order to manipulate the trap or to create a more traps.

In the recent years, a strong trend could be observed for miniaturization of optical traps, for example in order to use them in lab-on-chip setups^5^. Miniaturized optical traps do not feature large microscope objectives, but optical fibers. Apart from miniaturization, this trend results in a simplification of the optical setup, avoiding beam propagation in free space and avoiding the use of immersion oil. Further, it allows for manipulating particles anywhere in the solution. Optical fibers are also biocompatible, mechanically resistant and cheap. Several solutions are used in order to achieve a focused or a suitably formed beam, which involve modifying fiber ends: spherical tapering^6^ or sharp tapering of the fiber end^7^, inserting a lens or a sphere to the fiber end^8^, inserting an axicon lens^9^, inserting a spiral phase plate^10^, adding a Fresnel zone or phase plates^11^, annular exposure of the end^12^, and using a fiber with two cores^13^. The main drawback of such setups is either a very short manipulation distance, typically allowing to trap only the particles directly at the fiber tip, or obtaining a weakly focused two-dimensional optical trap due to the reduced numerical aperture (NA). To solve these problems graded index (GRIN) lenses can be used^14,15^.

In this paper, we present the modified stack-and-draw method that allows to fabricate a nanostructured GRIN lens with record large refractive index gradients. We present both a theoretical and an experimental study of a novel compact lensed fiber system utilizing the fabricated nanostructured GRIN lens. As a proof-of-concept of the new GRIN lensed fiber system, we demonstrate an experiment of an optical trapping of micrometer-sized glass beads. We also show that the proposed method for fabricating GRIN elements is compatible with optical fiber technology. Moreover, it allows for fabricating elements with any shape of the refractive index distribution in 2D. Thanks to that a new functionality could be achieved by replacing the GRIN lens with an axicon lens, vortex type elements, micro-lenses arrays, diffraction elements and others.

### Principles of the GRIN lens

The standard GRIN lens has a parabolic profile of the refractive index in a cross-section. The radial dependence of the refractive index is given by the equation:

1 $$n={n}_{g}(1-\frac{g\,{r}^{2}}{2})$$

where *g* is a gradient constant (units: mm^−2^), *r* is a distance from the optical axis (units: mm), and *n*_*g*_ is the index of refraction in the center of the element^16^. In this type of material, the focusing takes place in the bulk of the element and not on its surface. Because of this property the pitch remains constant, regardless of the environment of the element. The pitch Λ of a GRIN lens is the length of the distance between the consecutive focal planes, described by the following equation:

2 $${\rm{\Lambda }}=\frac{2\pi }{\sqrt{g}}$$

Depending on the length of the GRIN lens, we can design any imaging system which can be realized using the same index profile. The half-pitch element works as a lens, while the quarter-pitch element acts as the collimator for a point source located on the front surface of the lens.

GRIN lenses are currently produced from various types of materials: axial GRIN, radial GRIN and spherical GRIN materials^17,18^. The GRIN lens can also be fabricated with the use of different methods. The most common method for the fabrication of GRIN glass is ion exchange^19^, where it is possible to obtain *Δn* = 8 × 10^−2^, and ion stuffing^20^ where *Δn* = −6 × 10^−2^ at the radial distance of 250 µm. These methods have some limitations, with the most important disadvantages being very small contrasts of refractive index, only monotonic refractive index distribution^18^, and the fact that these methods often require the use of toxic ingredients. Also, the neutron irradiation technique can be used, where it is possible to obtain *Δn* = 2.28 × 10^−3^. However, it is limited due to the use of a neutron source and allows for fabricating structures with a monotonic change of refraction index^21^. The most significant change in the refraction index is achieved by modified chemical vapor deposition (MCVD), where typically it is possible to obtain *Δn* = 0.1 at the radial distance of 250 µm^22^. It is also possible to fabricate 1D multilayer GRIN with a refractive index gradient of *Δn* = 0.25 per 6.5 µm^23^. The disadvantage of this method is that it is time-consuming due to the multi-step process, but it allows to obtain any non-monotonic distribution of the refraction index.

Presently, various solutions, i.e. taper structures^24^, forming a focusing lens directly on the tip of an optical fiber^25^, or lens axicon^26^, are being tested in order to reduce the size of the optical elements so that they can be more easily integrated with other components, such as fibers. Nano-structuration and miniaturization of GRIN elements allows to integrate them with optical fibers and telecommunication systems. At the same time, it makes integration more difficult, because the size of such a lens is in the order of tens of micrometers^27^. Usually, the method is to glue or splice a glass element called spacer to the fiber, together with a piece of a gradient fiber, which plays the role of a lens^28^. This fiber system is usually called a “fiber probe”. The spacer separates the fiber and the lens and is used to ensure that the spot size of propagating light is the same as the diameter of the lens.

Mao *et al*.^29^ proposed using the fiber system for biomedical imaging. They performed experiments for two lengths of the fiber spacer and different types of GRIN fiber lenses of different core sizes and outer diameters. They measured the working distance (WD) between 80 ÷ 630 µm and the spot size, i.e. the beam diameter (BD) 13 µm for the GRIN lens of 90 µm in length, and 34 µm for the GRIN lens of 420 µm in length. Recently, Bi *et al*.^28^ examined theoretically and experimentally the GRIN fiber lenses of the length of 90 ÷ 120 µm. The measured working distances varied in the range of 500 ÷ 630 µm, and the spot diameters changed in the range of 23 ÷ 34 µm.

In both those reports, a relatively large BS is notable, ranging from several to a dozen of micrometers. This is due to the technological constraints associated with the fabrication of GRIN components and, consequently, the relatively small value of the constant gradient *g* in Eq. 1. Therefore, we propose a method which allows to significantly increase the constant g, from 14.14 mm^−2^ or 30.25 mm^−2^ (as in the abovementioned publications) to more than 80 mm^−2^. The modified stack-and-draw method potentially allows to fabricate the GRIN lens with constant gradient *g* over 17500 mm^−2^ for a 3 µm diameter GRIN element with the maximal refraction index in the center equal 1.5581, and the minimal refraction index equal 1.5273^27,30–32^. This would reduce the size of the BS to less than 1 µm. What is more, the proposed method is compatible with optical fiber technology and permits to fabricate a compact lensed fiber system utilizing the nanostructured GRIN lens. As a proof-of-concept of the technological novelty we demonstrate a fiber-nanostructured lens system used for two-dimensional optical trapping of dielectric elements.

## Results and Discussion

### Numerical analyzes

**Figure 1:**
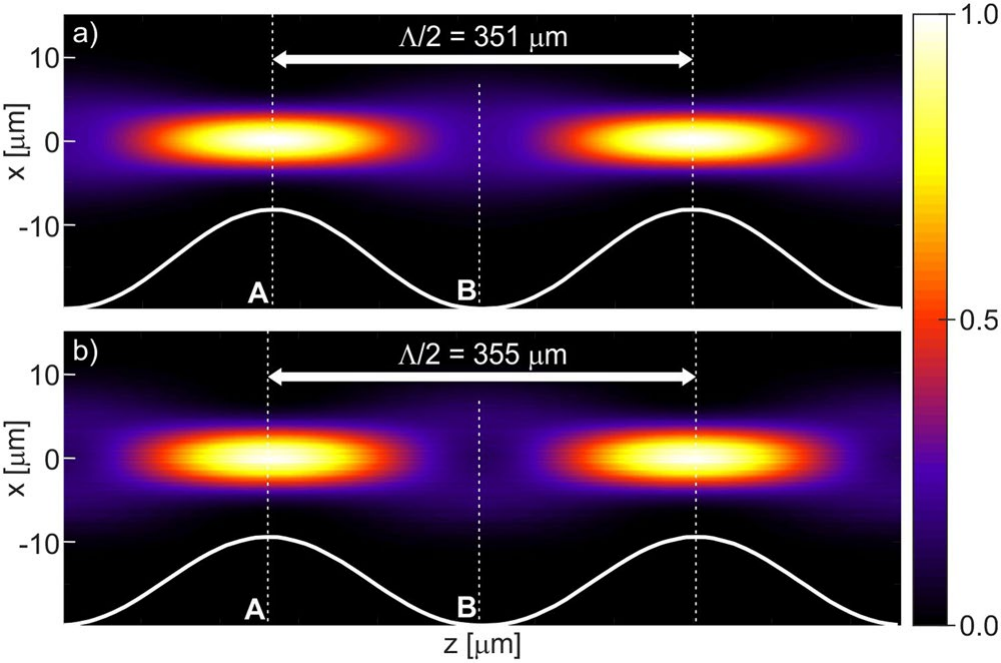
Normalized intensity of the beam propagation along the optical axis of an infinite GRIN lens for wavelength λ = 976 nm: (**a**) Ideal GRIN lens, (**b**) nGRIN lens. Planes A and B correspond to propagation distances in the lens, where the beam reaches the minimum and maximum width, respectively. Λ/2 denotes half-pitch length.

The theoretical part of the study consisted in running several numerical analyses. The main objective of the simulations was to compare the properties of (1) the nGRIN lens with the ideal GRIN lens, and (2) the lensed fiber element containing the nGRIN with a lensed fiber containing an ideal GRIN lens. In Fig. 1, we present simulations of light propagation through lenses of infinite length, for both the ideal GRIN lens and nGRIN lens. For the wavelength λ = 976 nm (used in all simulations and experiments), the results of those simulations show the half-pitch (Λ/2) length of the lens to be 351 ± 1 µm for the ideal lens, and 355 ± 1 µm for the nGRIN lens. This indicates a good correlation with the theoretical predictions, but for the nGRIN lens the half-pitch is longer, which suggests that the discretization of the gradient resulted in a lens with a slightly different gradient constant, or a slightly different refractive index distribution. This topic is discussed in more detail in the section on the nGRIN lens design.

**Figure 2:**
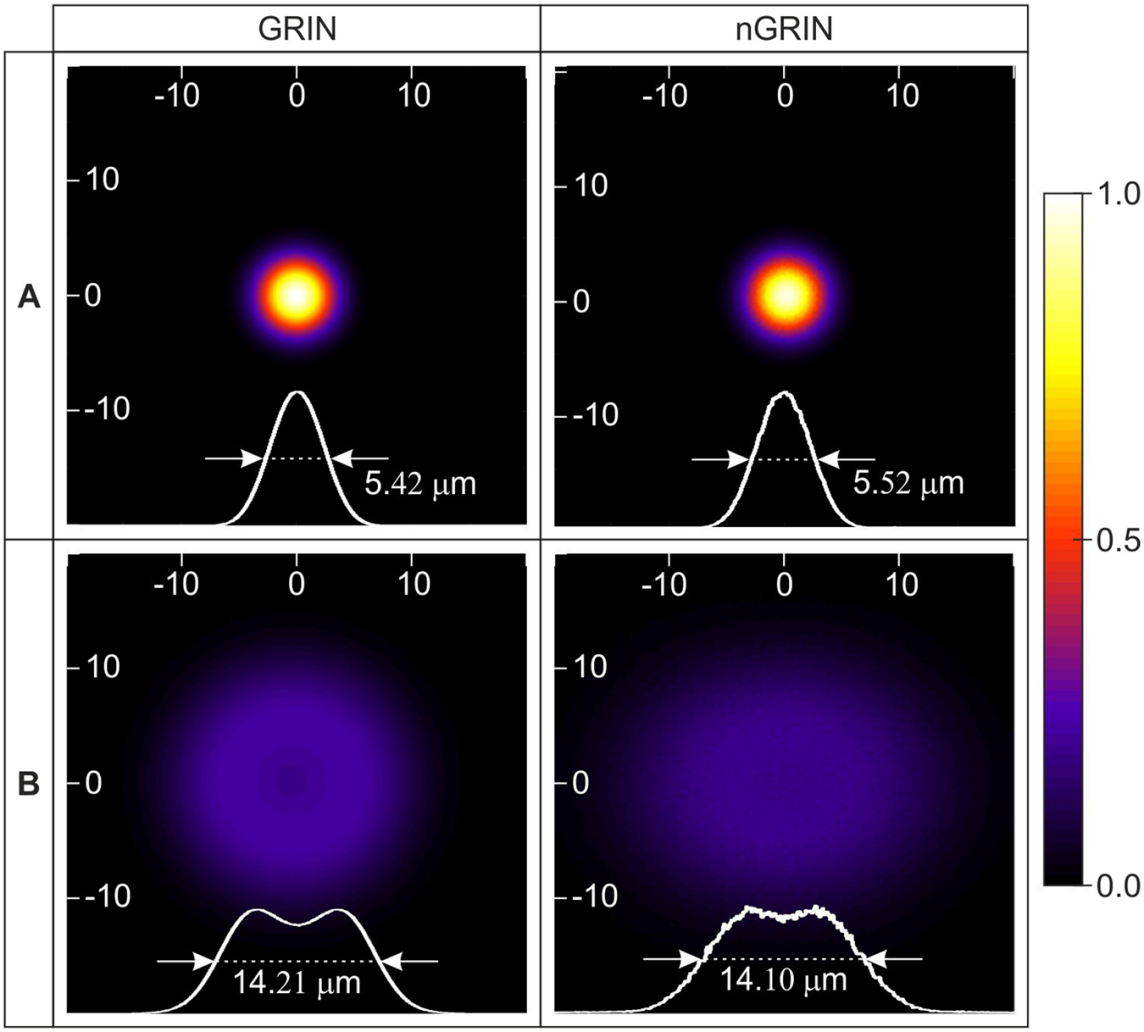
Comparison of light intensity distribution inside ideal GRIN and nGRIN lenses illuminated with a beam of FWHM equal 9.2 μm (Planes A and B denoted on Fig. 1).

In the consecutive simulations, we investigated the quality of the beam propagating inside the lens. The two-dimensional light intensity distributions in planes A, B marked on Fig. 1 are shown in Fig. 2. These results reveal a small discrepancy between the full width at half maximum (FWHM) values for the ideal lens and for the nGRIN lens. There are also differences in the distribution of light intensity, but their nature and size confirm that the nGRIN element can work as a lens.

**Figure 3:**
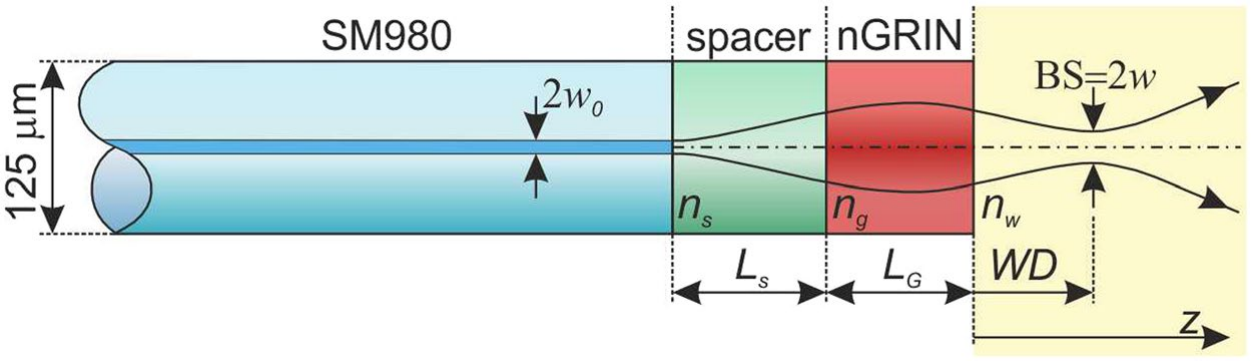
The scheme of the single mode nGRIN fiber lens system.

The next set of numerical analyses was performed for a lensed fiber element schematically shown in Fig. 3. The aim was to determine the optimum thickness of the spacer and the nGRIN lens for its use in an optical trapping setup, as discussed in the following section.

In the simulations, we assumed that the FWHM of the output beam from the single mode fiber equals 5.5 μm and the spacer was made of glass with the same refractive index as the glass in the center of the nGRIN lens (*n*_0_ = *n*_*g*_ = 1.57675 for λ = 976 nm). It was also assumed that a 976 nm wavelength would be used for optical trapping in water. The length of the spacer was chosen such as to ensure that all the nGRIN lenses with a diameter of 22.8 μm (defined as the diameter of the circle in which the nanostructure can be inserted) are used to transform the light coming out of the single mode fiber (SMF). The optimum spacer thickness was set at *L*_*s*_ = 105 μm. For shorter spacer lengths, the beam is not widened sufficiently, whereby only the central part of the lens is lighting. However, due to the limitations of technology, in its central part, the refractive index distribution is characterized by a constant value. For a longer spacer length, the incident beam will be too wide and the whole beam will not be focused by the lens.

**Figure 4:**
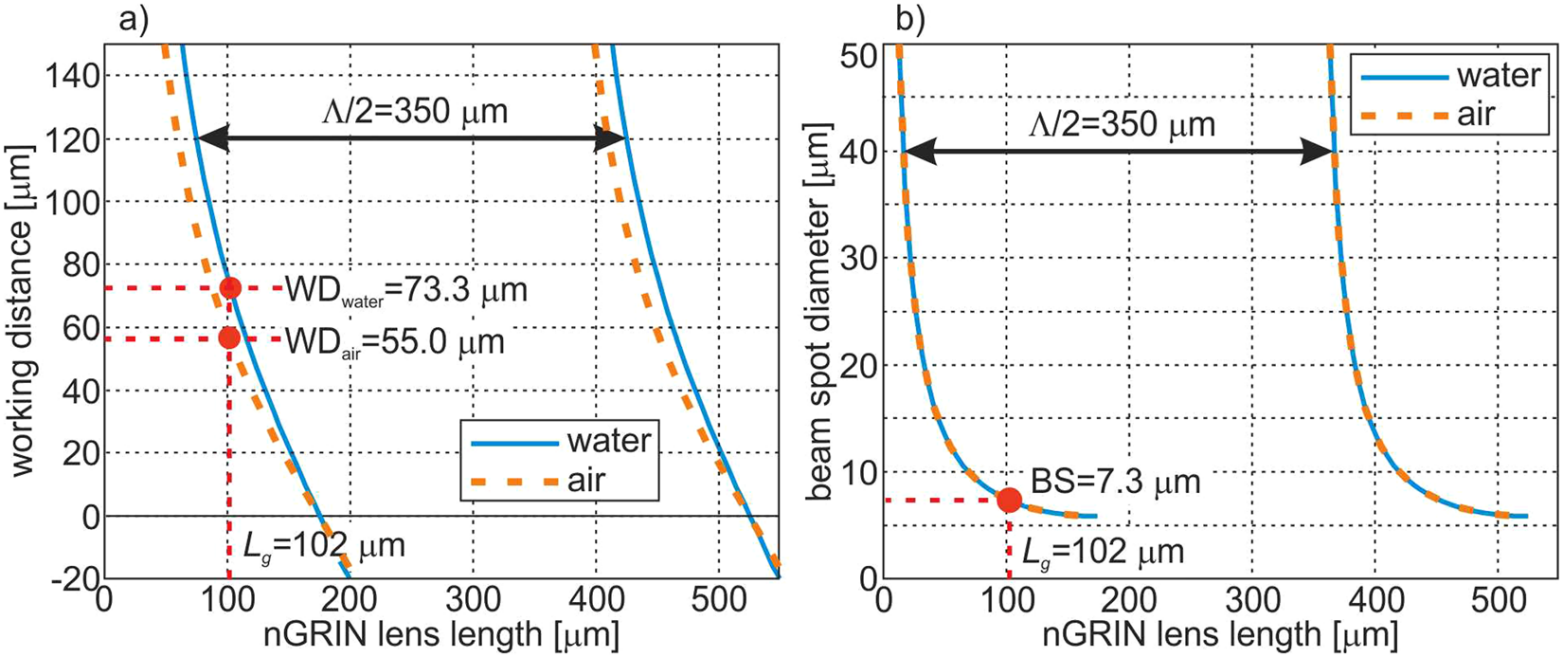
Dependence of the: (**a**) working distance (WD), and (**b**) beam spot diameter (BS) on thickness of the nGRIN lens for wavelength λ = 976 nm and immersed lensed fiber system in water and air. The red points denotes nGRIN lenses with a length of *L*_*G*_ = 102 μm.

The results achieved (Fig. 4) show that the minimal beam spot (BS) diameter is equal 5.8 μm and is obtained with 175 μm length of the nGRIN lens (the same BS diameter can also be obtained for the nGRIN lens of 175 + Λ/2 = 526 μm in length). Unfortunately, in this case the focus is on the end facet of the nGRIN lens, which means that the working distance (WD) equals zero. From the geometrical analysis of the optical trapping system based on the inverse microscope (described in the section: Optical trapping with the use of the nGRIN lens), and assuming the lensed fiber system is inclined at an angle of 45°, the resulting WD must be greater than 62.5 μm. Therefore, the nGRIN lens thickness was arbitrarily set to *L*_*G*_ = 102 μm. In this case BS is 7.3 μm and WD_water_ is 73.3 μm. Figure 4 also shows the results for an nGRIN lensed fiber system operating in the air. The results obtained show that the BS is the same for both systems working air and water. However, the working distance (WD) changes and WD_air_ is 55.0 μm. Magnification of our nGRIN lensed fiber system, defined as the ratio of the image size to the object size, is equal M = 1.33 for both environment: water and air.

### Experimental verification of the nGRIN lensed fiber system

**Figure 5:**
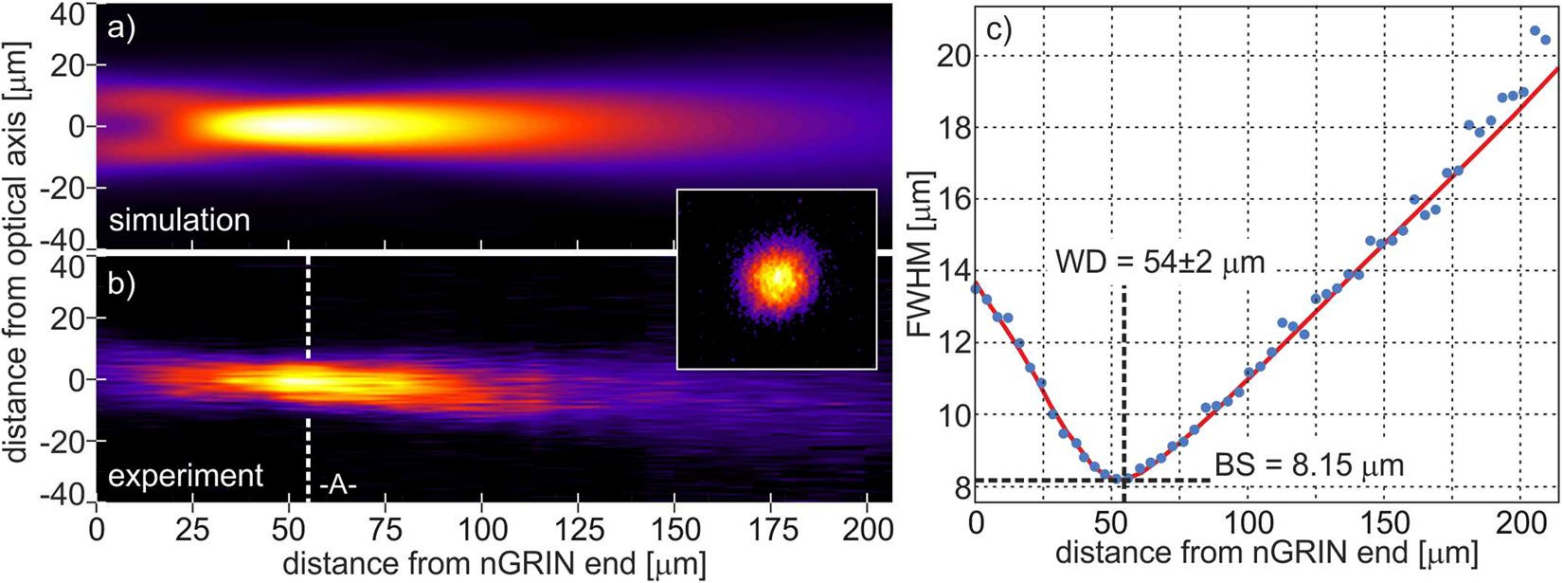
Beam propagation in air after the end plane of the *L*_*G*_ = 102 µm long nGRIN lens, with spacer *L*_*s*_ = 105 µm long. Normalized light intensity profile for: (**a**) simulation, and (**b**) experimental setup, (**c**) FWHM of the beam.

In the experimental part, we first fabricated the nGRIN lens with the use of the stack-and-draw method and then built the compact nGRIN lensed fiber system (see Materials and Methods). Next, the system was characterized to verify its working distance. Measurements were made in the air, and a 976 nm laser coupled into our lensed fiber system was used as the light source. We measured the beam size as a function of the distance from the nGRIN facet and estimated the working distance of the system, as 54 ± 2 µm (Fig. 5b), which is concurrent with the simulation results (Figs 4, 5a). At that distance, the FWHM of the focal spot is equal to 8.15 µm (Fig. 5c). This is slightly larger than the minimal spot size of 7.3 µm for a beam focused by such a lens, as calculated and presented in Fig. 4b.

### Proof of concept: Optical trapping with the use of the nGRIN lensed fiber system

**Figure 6:**
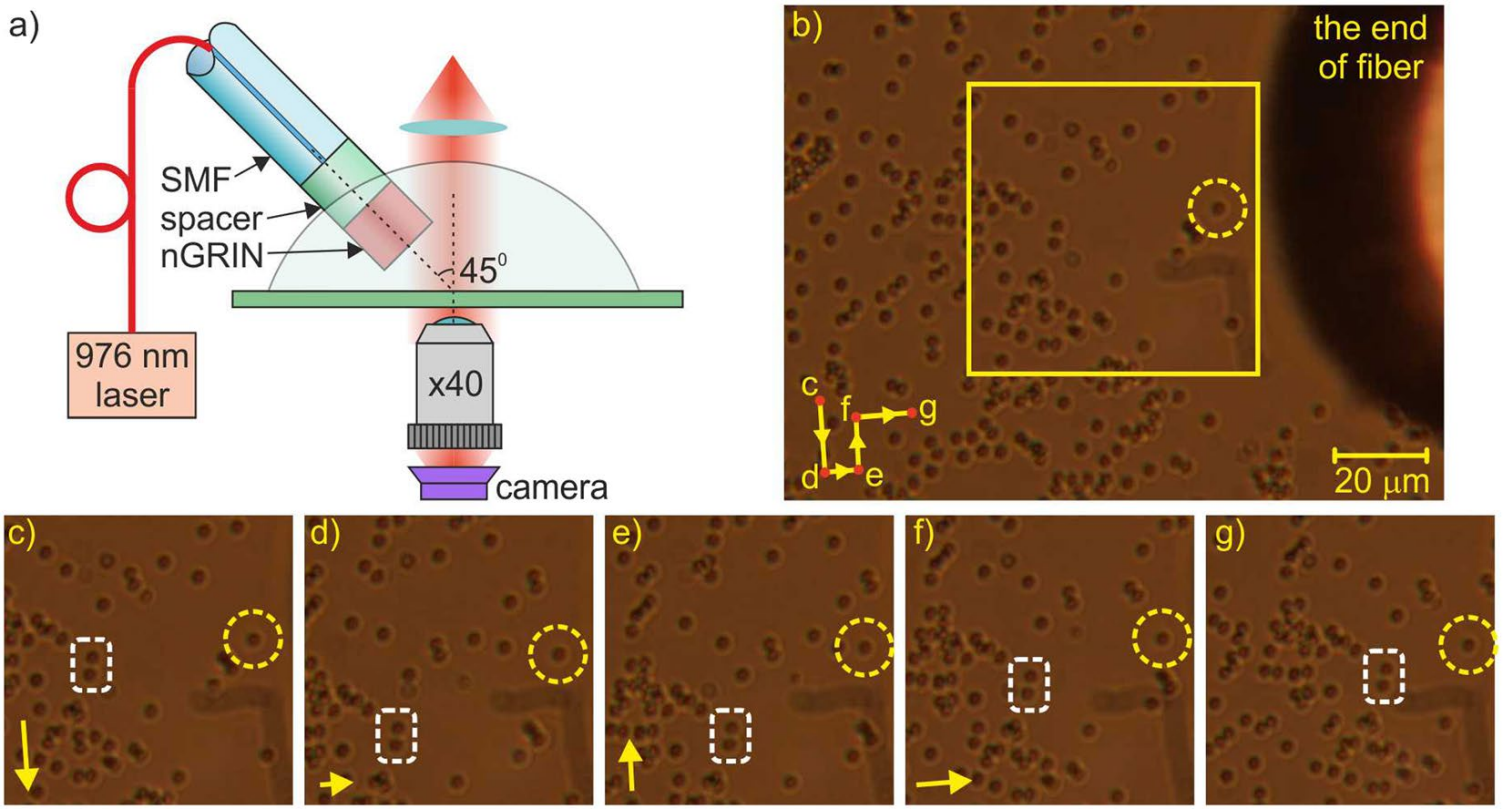
Demonstration of the 2D optical trapping of a single glass bead (results for 120 mW output light power): (**a**) Scheme of the trapping setup, (**b**) observed area (a sequence of movement of the trapped beads is presented in the lower left corner, letters indicate sequence of figures) (**c**–**g**), the trapped elements are surrounded by a yellow dashed line circle, two reference beads are surrounded by a white dashed line rectangle, and the yellow frame marks an enlarged field of observation), (**c**–**g**) subsequent positions of the silica beads (the arrows in the lower left-hand corner indicate the direction of movement of the trapped beads towards position indicated at the next figure). (Supplementary materials: Movie 1.avi).

**Figure 7:**
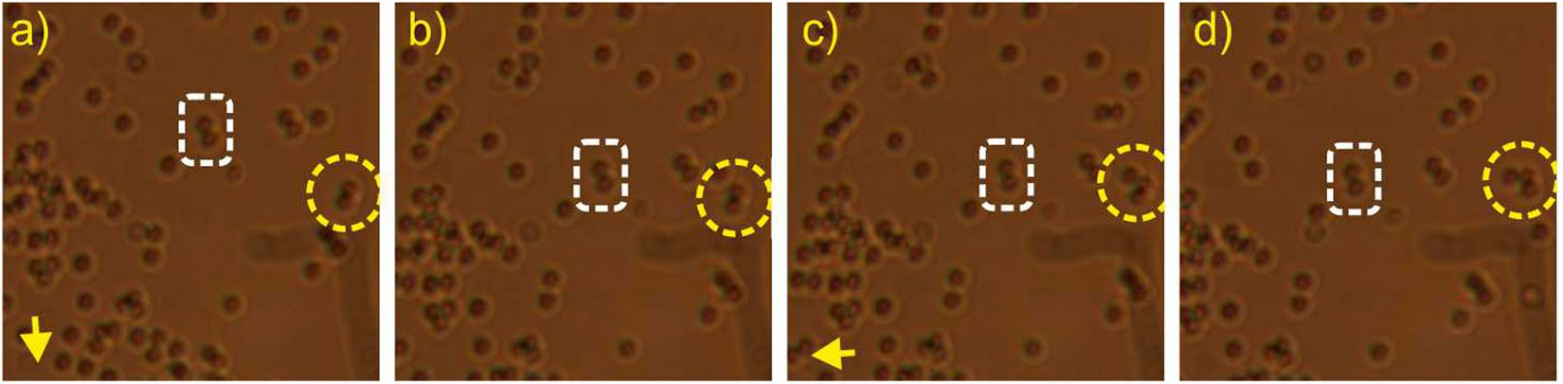
Simultaneous trapping (results for 120 mW output light power) of: (**a**,**b**) two glass beads, and (**c**,**d**) three beads (directions of movement of the trapped beads is indicated by an arrow at the lower left corner, the trapped elements are indicated with a yellow dashed line circle, two reference beads are indicated with a white dashed line rectangle). (Supplementary materials: Movie 2.avi).

In order to verify the applicability of the fabricated nGRIN lensed fiber system, we used it for 2D trapping, i.e. for pushing and pulling a single dielectric bead on the glass surface (Fig. 6). In the experiment, we utilized silica beads of 2 µm diameter immersed in water. Because the spot size was larger than 7 µm, which is larger than the diameter of a single silica bead being trapped, the proposed nGRIN lensed fiber system also trapped clusters of nearby beads. Figure 7 shows the examples of trapping of two and three glass beads simultaneously.

## Discussion

This paper presented the theoretical and experimental verification of a novel compact nGRIN lensed fiber system based on an nGRIN lens fabricated by the modified stack-and-draw technique. In the theoretical part, we showed how computational simulations supported the design of the nGRIN lens and the production process of the lensed fiber system. A high compatibility of the numerical analysis with the consecutive experimental results proves that it is possible to produce an nGRIN element according to the design and successfully use it in a fiber system.

We produced an nGRIN lens with numerical aperture NA = 0.16 and a parabolic distribution of the refraction index from two thermally matched glasses with gradient constant *g* greater than 80 mm^−2^ (22.8 μm nGRIN in diameter, *n*_*g*_ = 1.56895, *n* = 1.56078). This allowed for focusing light in the air at wavelength λ = 976 nm to a spot diameter of 8.15 μm, at a distance of 54 μm from the end facet of the nGRIN lens. In water, for the same wavelength, the beam was focused to a spot diameter of approximately 7.5 μm at a distance of 73 μm. The beam spot diameter obtained is sufficient to perform the trapping in 2D, which has been confirmed experimentally.

The lensed fiber system shows two main advantages of the nGRIN lens over conventional GRIN lenses. These are the small working distance and small beam spot diameter values, both stemming from the high value of gradient constant *g* of the nGRIN lens. Moreover, the characteristic of the nGRIN lens, which cannot be achieved with any other conventional method, was obtained in a relatively simple manner thanks to using the stack-and-draw technology. This indicates that lenses fabricated in such technology can potentially allow for optical trapping in 3D in a system with single GRIN lens.

The stack-and-draw method allows to fabricate elements with any shape of the refractive index distribution in the direction perpendicular to the length of the fiber, and in particular, diffraction elements and GRIN-type elements such as: elliptical lenses, axicons, micro-lenses arrays and optical vortex. Additionally, the elements obtained with this method are compatible with fibers optics, i.e. their size is comparable to standard fibers, so they can be combined into more complex optical systems such as the nGRIN lensed fiber system presented in this paper. The stack-and-draw method also allows for to combine several functionalities within a single fiber (e.g. a micro-lens, a drug delivery channel, electrodes), which can be useful in optofluidic applications. Moreover, by combining materials with appropriately selected dispersion properties the method allows to create nGRIN lenses whose performance is nearly independent on the wavelength. The stack-and-draw method also allows for a very high repeatability of the fabricated elements. First, a microstructured fiber is obtained characterized by identical parameters along its length, which is similar to the fabrication of standard optical fibers. Then, thousands of identical elements are obtained from one long fiber through cutting, which is cost-effective.

### Future research

**Figure 8:**
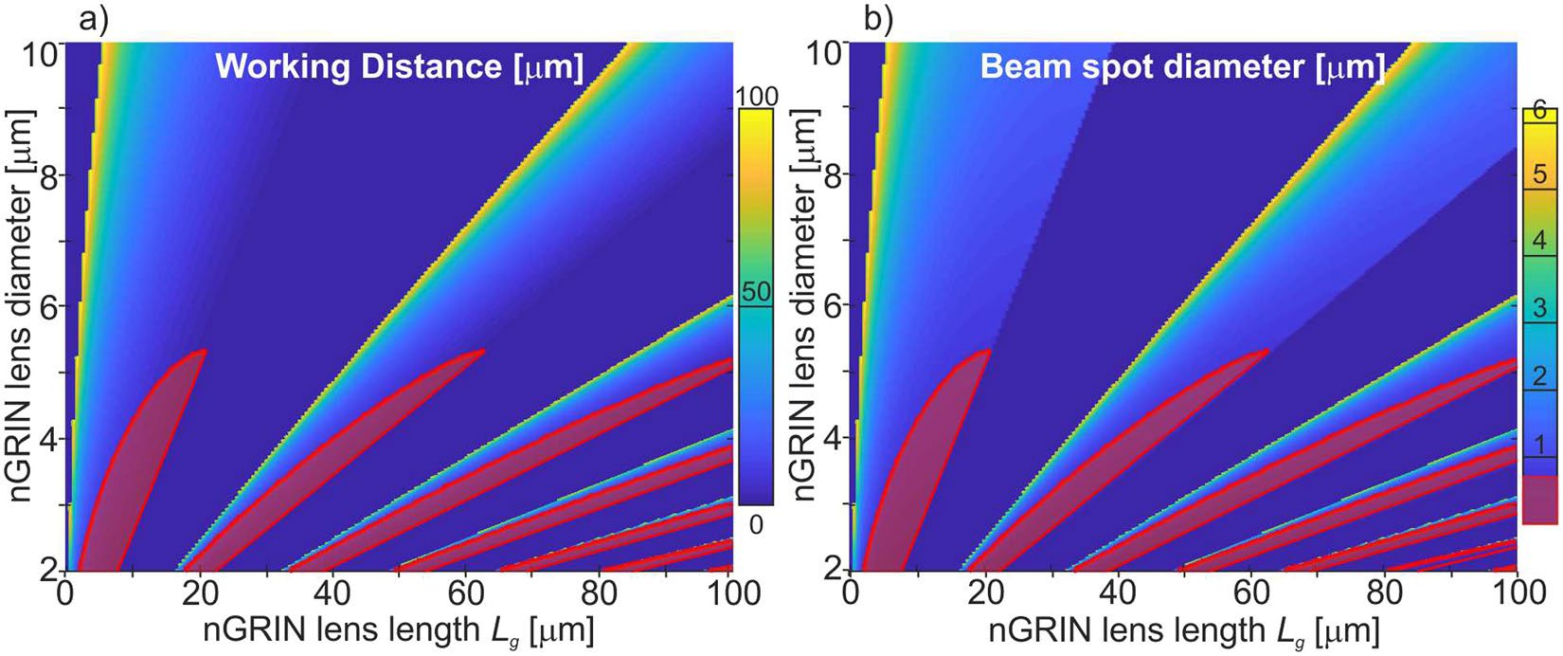
Relationship of the: (**a**) working distance (WD), and b) beam spot diameter (BS) on diameter and thickness of the nGRIN lens for wavelength λ = 976 nm and immersed lensed fiber system in water. For the red areas, BS is less than 0.7 μm.

As shown above, the beam spot equal to 7.3 µm enables optical trapping in 2D. The presented nGRIN lens, however, is insufficient to obtain the BS of less than 0.7 µm, which would be necessary for 3D stable traps^33^. However, since the nGRIN fabrication method presented in this work potentially allows to fabricate such a lens, thus, we assume that 3D optical trapping can be obtained in a system with a single GRIN lens. For this purpose, a lens with large gradient constant *g* is required. The way to do this is to increase the difference between the refractive index of the glass in the center and outside of the GRIN lens, and reduce the diameter of the lens. Buczyński *et al*.^32^ have shown that borosilicate soft glasses with the refraction index equal 1.5581 and 1.5273 can be used for this purpose. This work also shows the possibility of reducing the diameter of the nGRIN element to 3 μm. This, potentially, allows to fabricate GRIN lens with constant gradient *g* over 17500 mm^−2^ and to reduce the size of the BS to less than 0.7 µm. The analysis of how the working distance (WD) and the beam spot (BS) diameter depends on the diameter and length of the nGRIN lens is shown in Fig. 8.

Since the stack-and-draw method used for the fabrication of the nGRIN lens opens up the possibility of producing elements with any refractive index distribution, in further work, we plan to extend the scope of the use of fiber optic systems with new functionalities by replacing the nGRIN lens by: diffraction elements, axicons, micro-lenses arrays and optical vortex.

## Materials and Methods

### Design of the nGRIN lens

A nanostructured gradient index component consists of two different types of glasses which are assembled^34^ in a certain pattern, which results in the continuous refractive index distribution. The calculations for the distribution of the two glass types are based on the Effective Medium Theory (EMT)^35^, where two properties such as the conductivity δ and dielectric constant ε of the medium are taken under consideration. In this case, we need to describe the permittivity of the medium or the refractive index of the mixed materials. For this purpose, the Maxwell-Garnett (M-G) mixing formula for homogenized effective medium^28^ can be used. It defines the effective permittivity of the medium by averaging over a neighborhood around a certain point *r*, according to the formula^27,36^:

3 $${\varepsilon }_{eff}=\langle \varepsilon \rangle -f({U}_{r})\langle {\varepsilon }_{1}-{\varepsilon }_{2}\rangle \frac{{\varepsilon }_{1}-\langle \varepsilon \rangle }{3\langle \varepsilon \rangle }$$

where *ε*_1_ and *ε*_2_ are the permittivity of the two glasses, $\langle \varepsilon \rangle ={\varepsilon }_{1}-{\varepsilon }_{2}$ and *f*(*U*_*r*_) is the fill factor of *ε*_1_ in a certain neighborhood *U*_*r*_ around point *r*.

**Figure 9:**
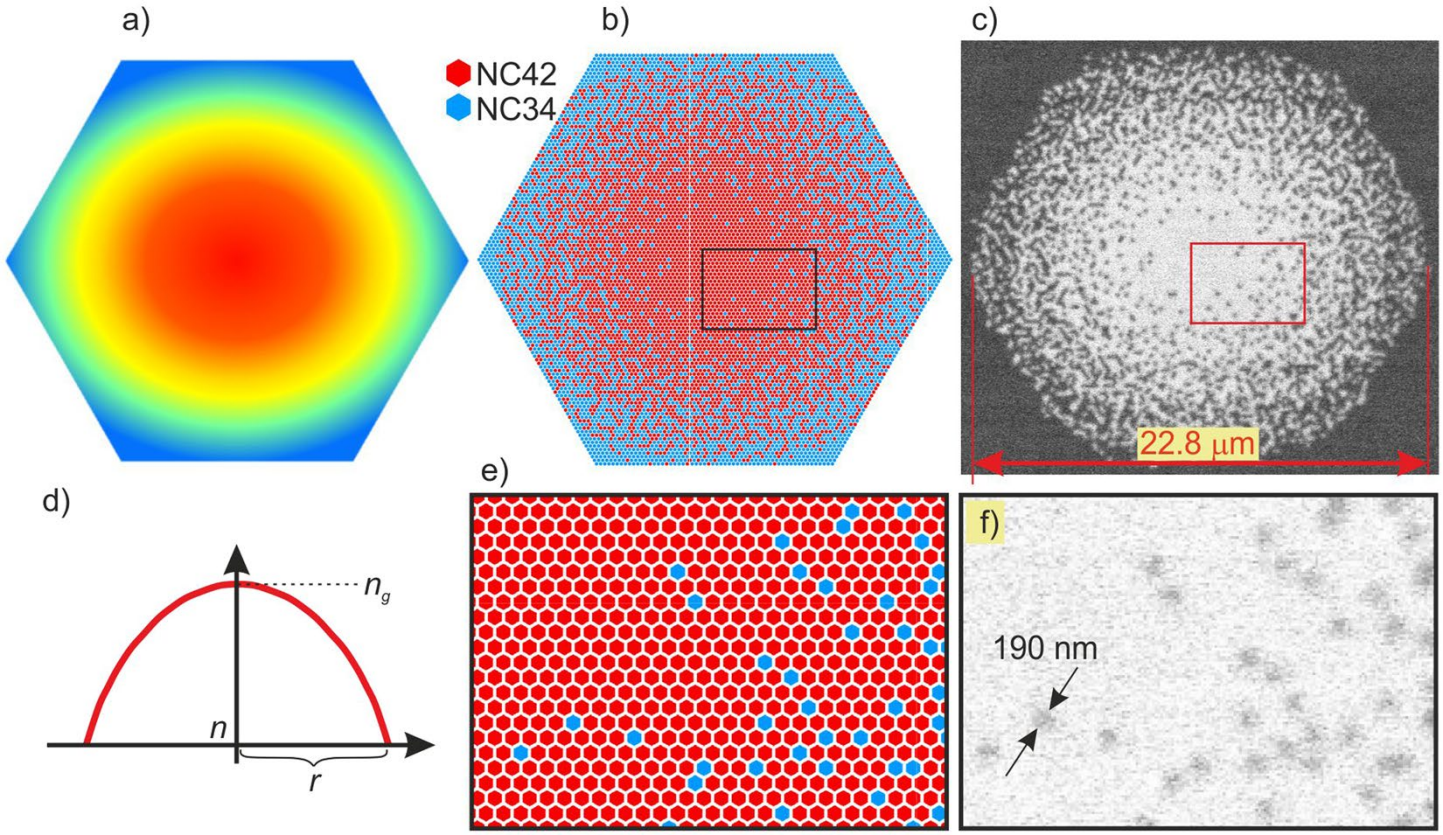
nGRIN lens: (**a**,**d**) ideal GRIN lens; (**b**,**e**) design of a preform composed of 9919 rods made from two glasses with refraction index *n*_*g*_ and *n*; (**c**) (detailed distribution of rods is shown in Supplementary materials), (**f**) SEM image of the final nGRIN structure.

**Figure 10:**
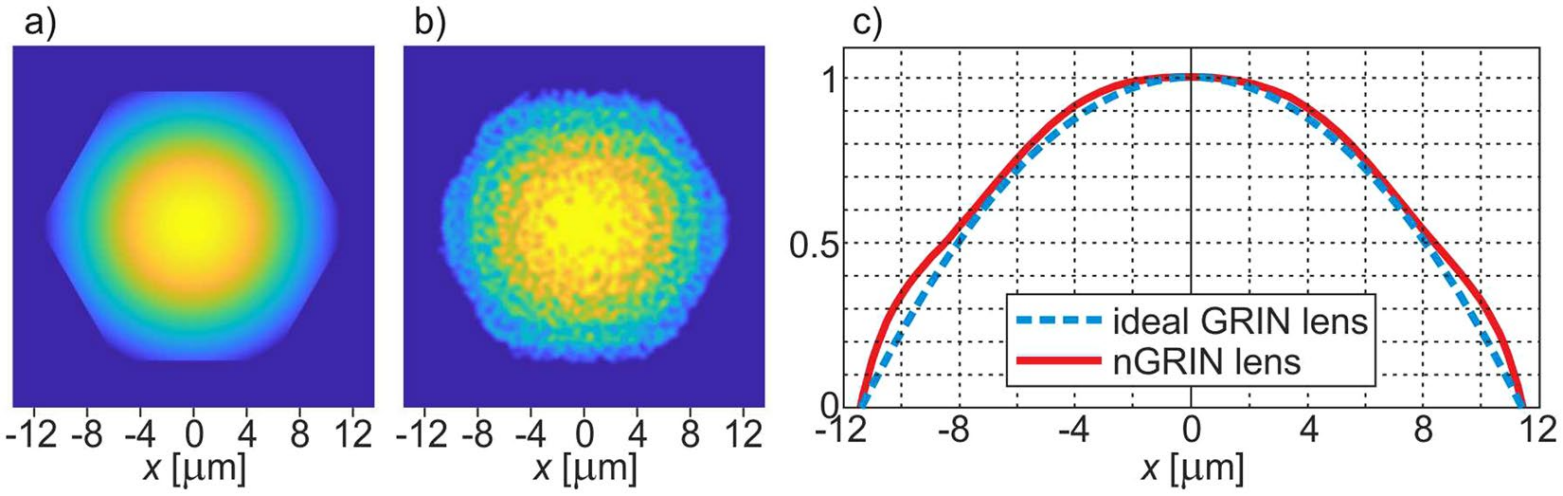
Refractive index distribution: (**a**) in the ideal lens, and (**b**) in the microstructural lens, (**c**) a comparison of the refractive index profiles for both lenses.

Technically, the nanostructured optical component consists of subwavelength sized glass rods what enables the heterogeneous materials (two different types of rods) to be considered as the homogeneous one^35^. Therefore the M-G mixing formula is accurate. The calculated pattern of the two glass rods is presented in Fig. 9b. The simulated annealing^37^ optimization process was used to change the continuous refractive index distribution to the discrete pattern of rods, corresponding to the GRIN lens with a parabolic distribution of the refractive index (Fig. 9a,d). Due to the finite number of rods, the average refractive index distribution did not perfectly reflect the distribution as in a lens with a continuous change in refractive index. Deviations from the ideal refractive index distribution are shown in Fig. 10. However, as shown in the simulation and experimental results, this did not significantly affect the performance of both GRIN lens and nGRIN lensed fiber system.

### Fabrication of the nGRIN lens

**Figure 11:**
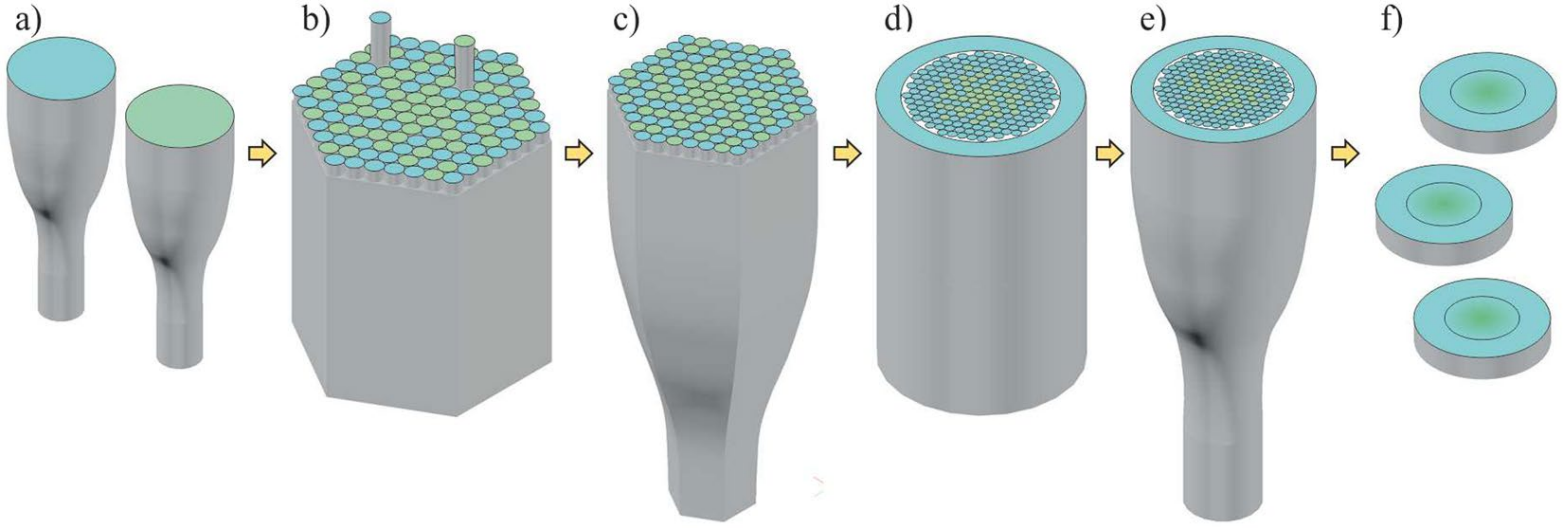
Scheme of the modified stack-and-draw technique: (**a**) glass rods preparation, (**b**) hexagonal preform stacked, (**c**) drawing hexagonal sub-preform, (**d**) surrounded preform by the glass rods, (**e**) drawing the final structure, (**f**) cut and polished final nGRIN elements.

The stack-and-draw process was used to develop the nGRIN microlens. The technique consists of several steps shown in Fig. 11.

**Figure 12:**
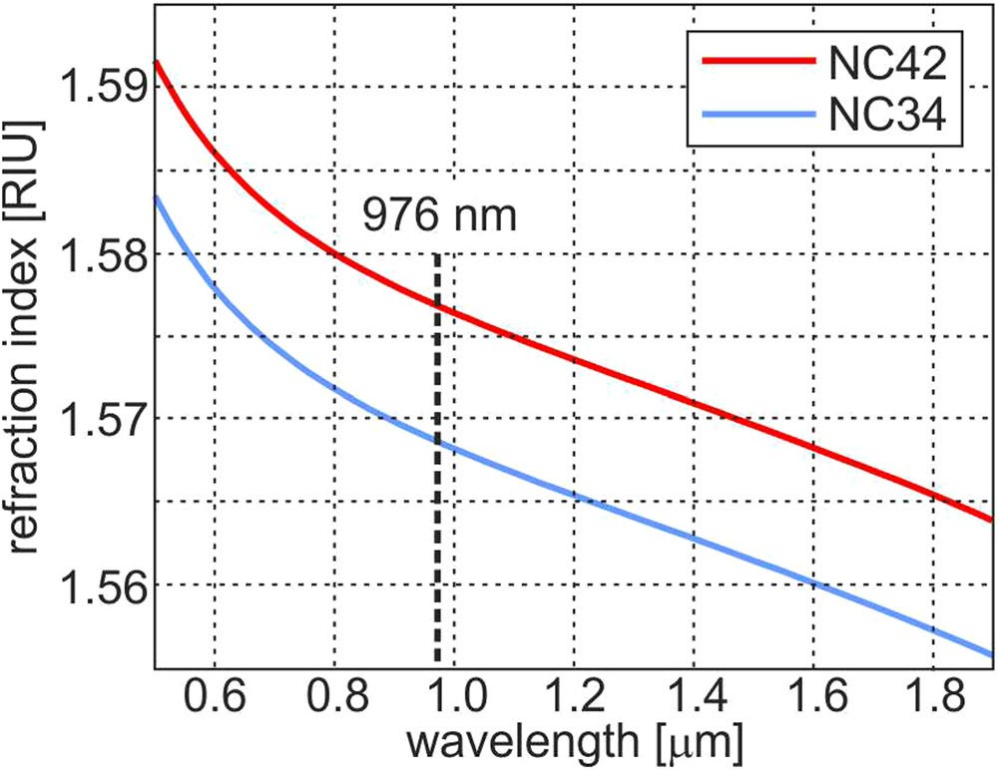
The refractive index as a function of the wavelength for the NC34 and NC42 borosilicate soft glasses.

First, we prepared round rods, approximately 0.5 mm in diameter, made from two types of borosilicate soft glasses (Fig. 11a) named NC42 (ng = 1.56895 for the sodium D line) and NC34 (n = 1.56078 for the sodium D line). The refraction index characteristics for glasses are shown in Fig. 12. Both glasses are thermally matched and have been successfully used previously^38,39^.

Next, the rods from both glasses were stacked to a hexagonal preform (Fig. 11b) according to the desired pattern (Fig. 9a) (detailed distribution of rods is shown in Supplementary materials). In this case, the preform consisted of 9919 rods (115 rods ordered on a diagonal, 5797 and 4122 rods with glass with higher and lower refraction index, respectively) and their pattern was chosen such that the average refractive index corresponded to the parabolic refractive index distribution in GRIN lens (Eq. 1). Subsequently, the preform was first drawn to a hexagonal structure of 3 mm in diameter (Fig. 11c). Next, the hexagonal sub-preform was surrounded by the glass rods of low index of refraction (Fig. 11d), to assure cylindrical shape of cladding and to facilitate the process of final element diameter scaling during the drawing process (Fig. 11e). In the last step, the fiber structures were cut into slices, grounded and polished to the required thickness (Fig. 11f).

In our final structures, the diameter of the individual rods (~190 nm) was equal or smaller than 1/5 of the propagating light wavelength (Fig. 9b).

The final nGRIN lens used for the lensed fiber system had the diameter of 22.8/125 µm, where the first value corresponds to the graded area diameter and the second value is the total fiber diameter. The gradient constant *g* is equal 80.14 mm^−2^ (Eq. 1) and the pitch Λ (Eq. 2) is equal 702 μm. The measured value of the numeric aperture NA = 0.16.

The stack-and-draw method allows to fabricate elements with unique properties. First of all, it allows to freely shape the distribution of the refractive index in the direction perpendicular to the length of the fiber. In particular, we have shown that we are able to fabricate with this technology diffraction elements (DOE)^38^ and GRIN-type elements such as: elliptical lenses^30^, lenses with an extended focal length – axicon^40^, micro-lenses array^41^ and optical vortex^42^, where the refractive index changes not along the fiber radius but perpendicularly. Moreover, the fiber with optical vortex structure cannot be fabricated with the use of any other methods available. Additionally, the elements obtained in this way are compatible with fibers optics, i.e. they have comparable size to standard fibers and, as shown in the article, can be combined into more complex optical systems such as the nGRIN lensed fiber system. The presented system allows for optical trapping in a single trap, but adding a DOE element or using an array of microlenses may allow to trap more elements. Similarly, a new functionality could be achieved by replacing the nGRIN lens with an extended focal length lens (axicon) or an element that allows the creation of an optical vortex.

Because the method is compatible with optical fiber fabrication, it is possible to combine several functionalities within a single fiber (micro-lens, drug delivery channel, electrodes), which can be useful in optofluidic applications. Moreover, stacking optical elements from two glasses and drawing them, allows to obtain elements with a very high gradient of the refractive coefficient, which, as described in the introduction, is not possible with other methods. The method described here is characterized by very high repeatability of fabricated elements. Similarly to the fabrication of optical fibers where a fiber with identical parameters along its length is obtained (with a good control of the drawing parameters), here the fiber structure with constant parameters is obtained. Then, from one long fiber thousands of identical elements are obtained through cutting.

The proposed method allows to create nGRIN lenses with performance nearly independent of the wavelength. These properties depend, to a large extent, on the chromatic properties of the glasses from which the GRIN element is fabricated. As opposed to the ion exchange method^19^, which does not grant full control over the chromatic properties of the fabricated elements due to the limited set of ions that can be exchanged, the method proposed here, allows to select glasses for their dispersive properties. In our case, the difference in the refractive index of NC34 and NC42 (Fig. 12), in the range from 0.5 to 2 μm, is at level of 10^−4^ RIU, which results in a very low dependence of the lens focal length on the wavelength.

### Fabrication of the nGRIN lensed fiber system

Usually, the splicing method is used to connect elements in fiber optics. However, in our case gluing was used due to the thermal mismatch between materials from which the fiber, spacer and nGRIN lens are made. The SM980 (Thorlabs) fiber was made of pure silica for which the glass-transition temperature is equal 1200 °C. The spacer and the lens were made of borosilicate soft glasses NC34 and N42, with a glass-transition temperature of 530 °C. Splicing of components made of materials with such a large thermal mismatch is challenging, however it can be done using a specialist splicing machine^43^.

**Figure 13:**
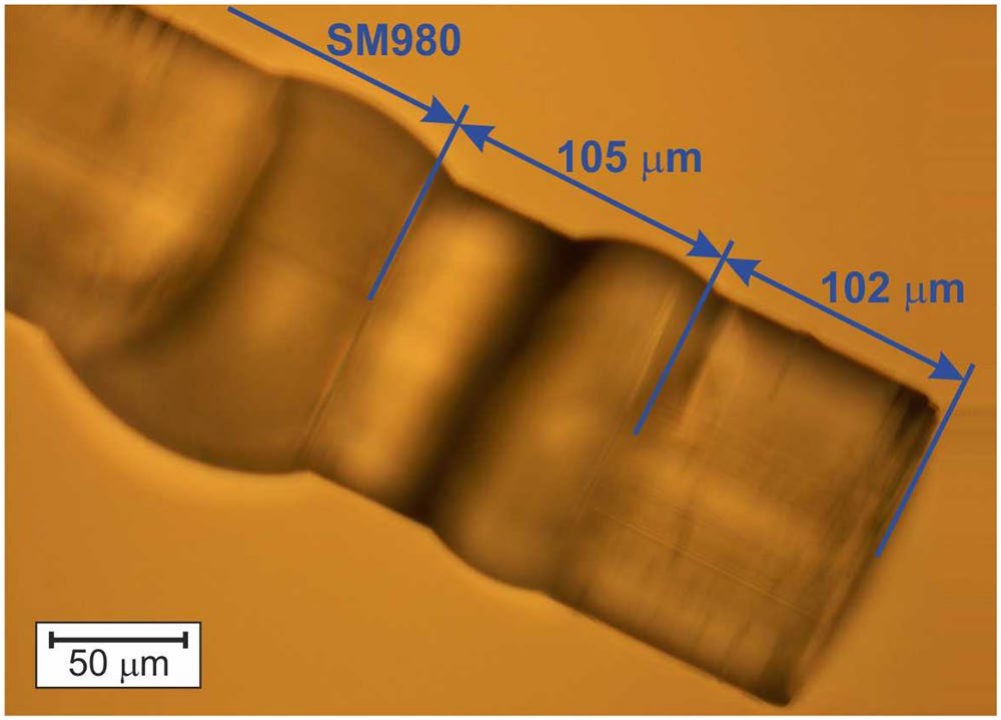
Microscopic image of 102 µm nGRIN lens, assembled with 105 µm spacer and SM980.

The nGRIN lensed fiber system consisted of a standard single mode fiber SM980 (Thorlabs) with diameter of 125 µm and a flat ending, and a bulk spacer of the same diameter attached to the fiber facet using optical glue whose refractive index was about 1.56. The nGRIN lens (125 µm in diameter) was also attached to the end facet of the spacer with glue (Fig. 13). The final nGRIN lensed fiber system allowed for focusing light in the air at wavelength λ = 976 nm to a spot diameter of 8.15 μm, at a distance of 54 μm from the end facet of the nGRIN lens. In water, for the same wavelength, the beam was focused to a spot diameter of approximately 7.5 μm at a distance of 73 μm.

The system was assembled in the following way. We began to attach the spacer and the nGRIN lens to the fiber by dipping the end of the fiber in the optical glue. The plate with the spacer was placed on the microscope table. Then, the fiber and the spacer were centered in one axis and placed adjacent to each other. The setup was illuminated with the UV light for several minutes to harden the glue. After the illumination, the same steps were executed with the nGRIN lens. After the second illumination, the probe was ready for measurements.

### Numerical simulations and calculations

Numerical simulations of the beam propagation both for the ideal and the nGRIN lenses were based on the fast Fourier transform beam propagation method (FFT BPM)^44^. The analysis was carried out for the Gaussian beam propagating at a distance of 400 µm in a conventional SM optical fiber with the core diameter of 4.6 µm. Then, the light passed through the bulk glass spacer of a fixed length (105 µm) and refractive index *n*_*S*_ = 1.56895, and then through the nGRIN lens. The two glasses used in the nGRIN structure of the lens had refractive indices of *n*_*g*_ = 1.56895 and *n*_0_ = 1.56078.

In order to ensure the convergence of the algorithm in the case of an ideal GRIN lens simulation, it is sufficient to sample every 0.25 μm in the plane perpendicular to the beam propagation and every 1 μm in the direction of the propagation. However, in the case of the nGRIN lens, it was necessary to sample with a resolution of 0.057 μm in the perpendicular plane and 0.2 μm in the direction of the beam propagation.

Calculations for the working distance (WD) and the beam spot diameter (BS) were based on the matrix formulation of Gaussian optics^29^ according to equations^45^:

4 $$WD=\frac{{n}_{w}[(1+{(\frac{{a}_{0}{L}_{s}}{{n}_{s}})}^{2}-{(\frac{{a}_{0}}{{n}_{g}g})}^{2})\sin (2g{L}_{g})-2\frac{{a}_{0}^{2}{L}_{s}}{{n}_{s}n{}_{g}g}\,\cos (2g{L}_{g})]}{2{n}_{g}g[{\sin }^{2}(g{L}_{g})+{(\frac{{a}_{0}}{{n}_{s}{n}_{g}g})}^{2}{({n}_{s}\cos (g{L}_{g})-{n}_{g}g{L}_{0}\sin (g{L}_{g}))}^{2}]}$$

5 $$BS=\frac{{a}_{0}{w}_{0}}{{n}_{g}g[{\sin }^{2}(g{L}_{g})+{(\frac{{a}_{0}}{{n}_{s}{n}_{g}g})}^{2}{({n}_{s}\cos (g{L}_{g})-{n}_{g}g{L}_{0}\sin (g{L}_{g}))}^{2}]}$$

6 $${a}_{0}=\frac{\lambda }{\pi {n}_{f}{w}_{0}^{2}}$$

where: *n*_*s*_ is the refractive index of the spacer between the optical fiber and the GRIN lens, *L*_*s*_ is the length of the spacer, *n*_*g*_ is the refractive index of the GRIN lens at the center, *L*_*g*_ is the length of the GRIN lens, *g* is the gradient constant of the GRIN lens, *n*_*w*_ is the refractive index of an imaged specimen, *w*_o_ is the initial beam radius from the optical fiber and, *n*_*f*_ is the refractive index of the optical fiber.

### Characterization of the nGRIN lensed fiber system

**Figure 14:**
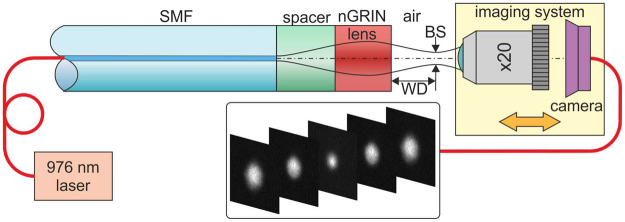
Schematic setup for measuring the working distance (WD) and the beam spot (BS).

The nGRIN lensed fibre system was characterized to verify its working distance (WD) and beam spot diameter (BS). All measurements were made in the air. We used 976 nm laser (BL976-SAG300. 300 mW) coupled into our lensed fiber system as the light source. At the output, the beam was magnified with an ×20 microscope objective (NA = 0.35) and then projected onto a CCD camera that works with a fixed gain in the linear regime (Fig. 14). The focal plane of the lens was determined by scanning the beam along the optical axis with the imaging system, with the translation resolution of ±50 nm. In contrast, the measurement of the absolute distance WD was made with an accuracy of ±2 μm.

### Optical trapping

**Figure 15:**
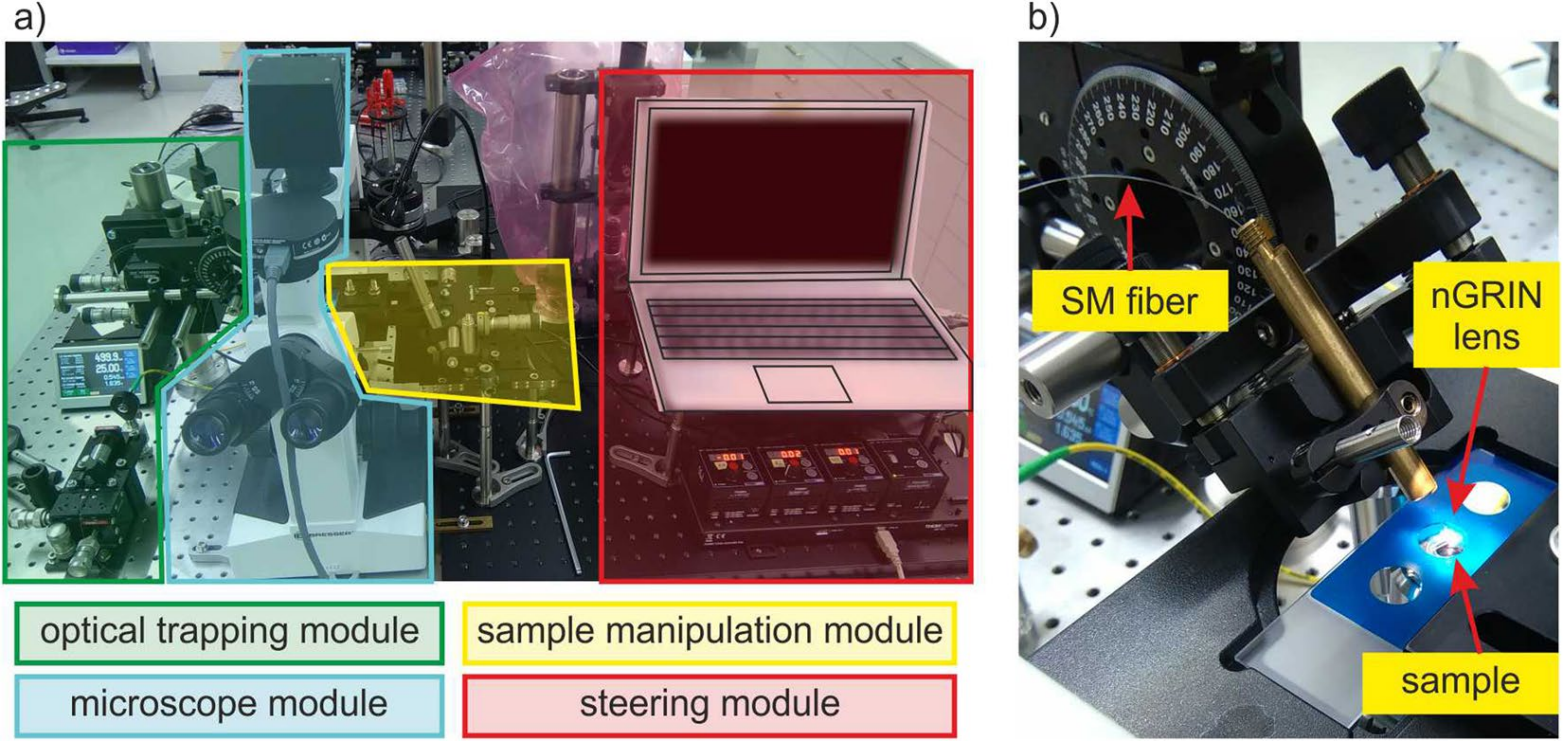
Optical trapping setup with nGRIN lensed fiber system: (**a**) the whole setup, and (**b**) enlarge trapping head.

To test the nGRIN lensed fiber system for optical trapping, the setup depicted in Fig. 15a was used. The setup featured a part dedicated to imaging and image acquisition (tinted blue in Fig. 15a), a part devoted to building the optical trap (tinted green in Fig. 15a), and a part allowing for manipulating the sample (tinted red in Fig. 15a). The image acquisition system was based on the inverted microscope with an ×40, NA = 0.65 objective, and a CMOS camera (SC50), before which a dichroic filter was located. A precise manipulation of the sample was possible thanks to a 3-axis piezo micromanipulator with 20 μm range of motion. The optical fiber with a glued nGRIN lens was placed in a metal holder and fixed on a computer-steered rotating table which allowed for precisely setting the tilt angle of the fiber (Fig. 15b). A 976 nm pigtailed laser diode was used for the trapping (BL976-SAG300. 300 mW) wherein the measured light power at the nGRIN lensed fiber output was 120 mW.

A modular structure of the setup allows for its simple integration with the existing inverted microscope systems. Moreover, joining the sample shifting module with the optical trapping system makes the setup independent from the microscopic system. The setup does not use any microscope optical path and can be added to the majority of microscopes.

## Electronic supplementary material

Supplementary materials

Movie1

Movie 2
