# Supplementary material for: Integrating Free-Form Nanostructured GRIN Microlenses with Single-Mode Fibers for Optofluidic Systems
Source: Sci Rep. 2018 Mar 22;8:5072. doi: 10.1038/s41598-018-23464-6 (PMC5864828; doi:10.1038/s41598-018-23464-6)

Integrating Free-Form Nanostructured GRIN Microlenses with Single-Mode Fibers for Optofluidic Systems

Rafał Kasztelanic,^1,2^ Adam Filipkowski,^1^ Alicja Anuszkiewicz,^1^ Paulina Stafiej,^1,2^ Grzegorz Stepniewski,^1^ Dariusz Pysz,^1^ Konrad Krzyżak,^1^ Ryszard Stepien,^1^ Mariusz Klimczak,^1^ and Ryszard Buczynski^1,2*^

^1^Department of Glass, Institute of Electronic Materials Technology, Wolczynska 133, 01-919 Warsaw, Poland

^2^Faculty of Physics, University of Warsaw, Pasteura 7, 02-093 Warsaw, Poland

*Corresponding author: [ryszard.buczynski@itme.edu.pl](mailto:ryszard.buczynski@itme.edu.pl)

**Supplementary materials**

**Supplementary Video 1:**

Movie1.avi – Demonstration of the 2D optical trapping of a single glass bead.

**Supplementary Video 2:**

Movie2.avi – Demonstration of the 2D optical trapping of two and three glass beads.

**Supplementary Figure 1:**

Detailed distribution of rods from two types of glass in the nGRIN lens.


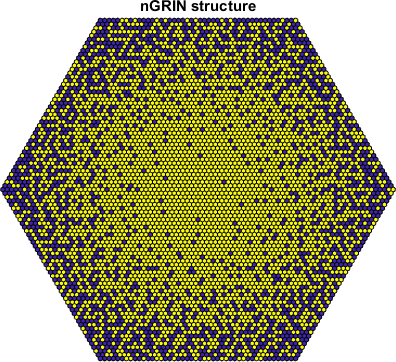

Supplement: Supplementary file 1 — Supplementary materials [file 41598_2018_23464_MOESM1_ESM.doc]
